# Forward genetics identifies a novel sleep mutant with sleep state inertia and REM sleep deficits

**DOI:** 10.1126/sciadv.abb3567

**Published:** 2020-08-12

**Authors:** Gareth T. Banks, Mathilde C. C. Guillaumin, Ines Heise, Petrina Lau, Minghui Yin, Nora Bourbia, Carlos Aguilar, Michael R. Bowl, Chris Esapa, Laurence A. Brown, Sibah Hasan, Erica Tagliatti, Elizabeth Nicholson, Rasneer Sonia Bains, Sara Wells, Vladyslav V. Vyazovskiy, Kirill Volynski, Stuart N. Peirson, Patrick M. Nolan

**Affiliations:** 1 Mammalian Genetics Unit, MRC Harwell Institute, Harwell Science and Innovation Campus, Oxfordshire, UK.; 2 Sleep and Circadian Neuroscience Institute (SCNi), Nuffield Department of Clinical Neurosciences, University of Oxford, Oxford, UK.; 3 UCL Queen Square Institute of Neurology, University College London, London, UK.; 4 Mary Lyon Centre, MRC Harwell Institute, Harwell Science and Innovation Campus, Oxfordshire, UK.; 5 Department of Physiology, Anatomy and Genetics, University of Oxford, Oxford, UK.

## Abstract

Switches between global sleep and wakefulness states are believed to be dictated by top-down influences arising from subcortical nuclei. Using forward genetics and in vivo electrophysiology, we identified a recessive mouse mutant line characterized by a substantially reduced propensity to transition between wake and sleep states with an especially pronounced deficit in initiating rapid eye movement (REM) sleep episodes. The causative mutation, an Ile102Asn substitution in the synaptic vesicular protein, VAMP2, was associated with morphological synaptic changes and specific behavioral deficits, while in vitro electrophysiological investigations with fluorescence imaging revealed a markedly diminished probability of vesicular release in mutants. Our data show that global shifts in the synaptic efficiency across brain-wide networks leads to an altered probability of vigilance state transitions, possibly as a result of an altered excitability balance within local circuits controlling sleep-wake architecture.

## Teaser

A noninvasive sleep screen uncovers a *Vamp2* mouse mutation with synaptic deficits underlying substantial sleep disturbances.

## INTRODUCTION

Despite advances in our understanding of sleep neurophysiology (*1*, *2*), the genetic regulation of the fundamental vigilance states—wakefulness, non–rapid eye movement (NREM) sleep, and REM sleep—remains poorly understood. A subcortical circuitry of neuromodulatory nuclei is thought to regulate global sleep-wake transitions, but the specific role of individual components remains to be determined. While high-throughput forward genetics screening has provided invaluable insights into the molecular genetic mechanisms underlying circadian rhythms (*3*–*5*), traditional electroencephalographic (EEG) methods of studying sleep are not conducive to high-throughput approaches and, currently, only a single study using such an approach has been published (*6*). Here, we adopted a high-throughput hierarchical approach, initially using behaviorally defined sleep before EEG/electromyography (EMG) to identify mutant pedigrees with abnormal sleep-wake parameters in *N*-ethyl-*N*-nitrosourea (ENU) G3 pedigrees. Cloning and sequencing of the strongest phenodeviant pedigree identified a missense mutation in the transmembrane domain of VAMP2, the core vSNARE protein mediating synaptic vesicle fusion and neurotransmitter release. EEG analysis confirmed the reduced sleep phenotype and revealed a marked decrease in REM sleep. Most notably, while vigilance states still could be reliably determined based on EEG and EMG signals, VAMP2 mutant animals showed a profound deficit in their capacity to switch states of vigilance once a specific state had been initiated. To determine how such a previously unexplored phenotype may arise, we show, using cellular, molecular, imaging, and electrophysiological studies, that vesicular release efficiency and short-term plasticity are drastically affected in mutants. The consequences of this deficit in neuronal firing and the inherent inertia in state switching demonstrate a hitherto uncharacterized role for VAMP2 in sleep and highlight how new aspects of gene function, even for well-characterized genes, continue to be uncovered using forward genetics.

## RESULTS

### The *Vamp2^rlss^* mutation is associated with sleep deficits in a forward genetics screen

**Fig. 1:**
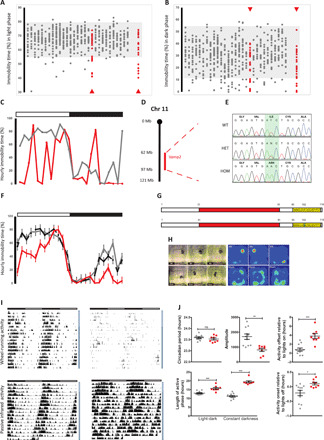
ENU sleep duration screen identifies a G3 pedigree with a mutation in *Vamp2*. Percent immobility-defined sleep during light (**A**) and dark (**B**) phases. Individuals from G3 pedigrees are shown in columns. Shaded areas represent normal immobility range. Arrowheads indicate the first and second cohorts of the pedigree. (**C**) Percentage hourly immobilities for an affected individual (red) and unaffected littermate (black). Light (open bar) and dark (filled bar) phases are indicated above the graph. (**D**) Mapping of affected individuals identified a 35-Mb region on Mmu11 including the *Vamp2* locus. (**E**) Whole-genome sequencing identified a single mutation in affected animals, resulting in an Ile102Asn substitution in the transmembrane region (yellow) of VAMP2 (**G**); SNARE motif shown in red. (**F**) Hourly immobility percentages in homozygotes (red), heterozygotes (black dashed), and wild types (WT) (black). (**H**) Sleep patterns from video stills and heat maps over the duration of the light phase in WT (top panels) and homozygotes (bottom panels). (**I**) Double-plotted actograms of WT (left panels) and homozygotes (right panels). Top panels show wheel-running activity, and bottom panels show movement using passive infrared (PIR) sensors. Light and dark horizontal bars represent periods of light and dark where appropriate. Vertical bars to the right represent periods of 12:12 light dark (white) or constant darkness (gray). (**J**) Circadian measures in WT (gray) and homozygotes (red) calculated from PIR data. Individual data points are shown as means ± SEM, *P < 0.05, **P < 0.01, ***P < 0.001. ns, not significant.

To identify mouse lines with sleep deficits, we used home-cage video tracking to measure immobility-defined sleep (*7*) in cohorts from G3 pedigrees generated in a large ENU mutagenesis program (*8*). By plotting percentage time spent immobile during light and dark phases (Fig. 1, A and B), we identified a pedigree, called restless (*rlss*, MGI:5792085), where multiple individuals expressed reduced immobility. Differences were particularly evident during the light phase and were confirmed by screening a second cohort from the same pedigree. Further analysis in 1-hour time bins indicated a strong effect throughout the first 8 hours of the light phase and toward the end of the dark phase (Fig. 1C). Using DNA from affected individuals, we mapped the nonrecombinant mutant locus to a 35-Mb region on chromosome 11 (Fig. 1D). Whole-genome sequencing revealed a single high-confidence coding sequence mutation within the nonrecombinant region. Sequence validation in multiple affected individuals consistently identified a single coding sequence variant cosegregating with the mutant phenotype, a T441A transversion in *Vamp2* (*Syb2*) resulting in an Ile102Asn substitution in the protein’s transmembrane domain (Fig. 1, E and F). VAMP2 is the major neuronal vesicular component of the soluble *N*-ethylmaleimide–sensitive factor (NSF) attachment protein receptor (SNARE) complex, fundamental to neurotransmitter exocytosis. Outcrossing affected individuals to wild-type (WT) mates confirmed the recessive phenotype, with immobility-defined sleep in heterozygotes being no different to WT. Statistical analysis of immobility in 1-hour bins confirmed a significant genotype effect and pairwise comparisons confirmed that sleep in homozygotes was significantly reduced in the first 7 hours of the light phase and the final 7 hours of the dark phase (Fig. 1F; for this and all other statistical analysis, refer to table S1). Further analysis of mutants also identified unusual sleep behaviors, where individuals showed no preference for nesting in sheltered parts of the home cage, frequently moved sleep position within the cage, and assumed atypical sleep postures (Fig. 1H). Conventional circadian parameters using cages with wheels could not be reliably measured in homozygotes (Fig. 1I). However, passive infrared monitoring of circadian rhythms (*9*) demonstrated that the homozygote circadian period was no different from controls, while period amplitude and activity parameters were affected (Fig. 1, I and J).

### Reduced vigilance state transitions and a pronounced deficit in REM sleep are prominent EEG anomalies in *Vamp2^rlss^* mice

**Fig. 2:**
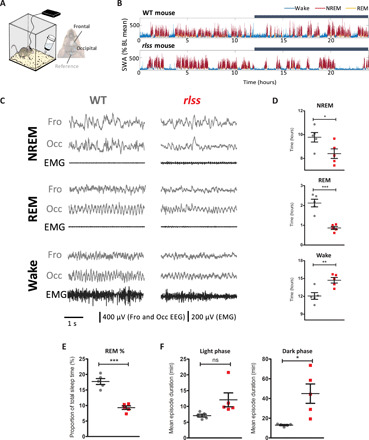
EEG abnormalities in *Vamp2^rlss^*. (**A**) A tethered EEG setup was used to perform chronic recordings from the frontal and occipital cortex. (**B**) Slow-wave activity (SWA) levels across 24 hours in individual WT and *Vamp2^rlss^* mice. SWA levels are expressed as percentage of the 24-hour baseline (BL) mean. Color coding indicates vigilance states. Dark phase is indicated with a dark bar above the graphs. (**C**) Representative EEG and EMG traces across vigilance states from individual WT and *Vamp2^rlss^* mice. (**D**) Time spent in each vigilance state over 24 hours for WT (gray) and *Vamp2^rlss^* (red) mice. (**E**) Percentage of total sleep time spent in REM sleep in WT (gray) and *Vamp2^rlss^* (red) mice. (**F**) Mean duration of wake episodes for light and dark phases in WT (gray) and *Vamp2^rlss^* (red) mice. Individual data points are shown, as is mean ± SEM, *P < 0.05, **P < 0.01, ***P < 0.001.

To investigate sleep-wake architecture in *Vamp2^rlss^* mice, we implanted cortical EEG and nuchal EMG electrodes (Fig. 2A) and performed 24-hour baseline home-cage sleep recordings (Fig. 2B). EEG/EMG signatures were generally typical of wakefulness, NREM, and REM sleep (*10*), although more pronounced slow-wave activity (0.5 to 4 Hz) during NREM sleep and slower theta oscillations during REM sleep were noted in *Vamp2^rlss^* (Fig. 2C and fig. S1A). However, over the entire 24-hour period, *Vamp2^rlss^* mice accumulated 1.4 hours (14%) less NREM sleep and 1.2 hours (57%) less REM sleep, with a ratio of NREM sleep time per wake time significantly reduced (Fig. 2D). Plotting the distribution of vigilance states across 24 hours showed the difference between genotypes was especially apparent for REM sleep (figs. S1B and S2A), a conclusion supported by a decreased REM–to–total sleep ratio (Fig. 2E), while the decrease in NREM sleep during the dark period was partially compensated during the second half of the light phase (figs. S1B and S2A).

**Fig. 3:**
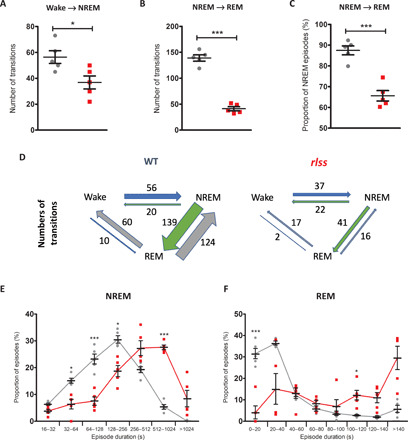
Vigilance state transitions in *Vamp2^rlss^*. WT shown in grey; rlss shown in red. (**A**) Number of wake-to-NREM transitions across 24 hours. (**B**) Number of NREM-to-REM transitions over 24 hours. (**C**) Percentage of NREM-to-REM transitions (normalized against total number of NREM episodes) over 24 hours. (**D**) Schematic indicating differences in number of wake-to-NREM and NREM-to-REM transitions over 24 hours. For (A) to (D), all NREM and wake episodes of at least 1 min within a 24-hour baseline day were included (no minimum duration for REM episodes). (**E**) Distribution of NREM episode durations over 24 hours. Here, all NREM episodes longer than 16 s are included. (**F**) Distribution of REM episode durations over 24 hours. Individual data points are shown, as is mean ± SEM, *P < 0.05, ***P < 0.001.

Increased wakefulness and longer wake episodes were evident in homozygotes (Fig. 2, D and F, and fig. S2B), suggesting an increased wake state continuity. In considering all wake episodes other than brief awakenings (≤16 s), their frequency was halved in *Vamp2^rlss^* mice, suggesting that once wakefulness is initiated, it is sustained for longer durations. Investigations as to whether reductions in sleep are related to a reduced probability of state transitions showed that the number of wake-to-NREM transitions was markedly reduced in homozygotes (Fig. 3A). Similar dynamics also manifested in state transitions within sleep; once an NREM sleep episode was initiated, it was less likely to transition into REM sleep (Fig. 3, B and D). Almost 90% of all NREM episodes terminated in REM sleep in WT mice, compared with less than 70% in *Vamp2^rlss^* mice (Fig. 3C). This indicates that, along the continuum of wake→NREM→REM occurrences, the inertia to transition to the next state is progressively increased in *Vamp2^rlss^* mice (Fig. 3D). Consistently, the distribution of episode durations for NREM and REM sleep showed an increased incidence of longer episodes for both sleep states in *Vamp2^rlss^* mice (Fig. 3, E and F), further suggesting that once a specific state is initiated, it is less likely to terminate.

### Neuronal structural and synaptic changes are evident in *Vamp2^rlss^* mice

**Fig. 4:**
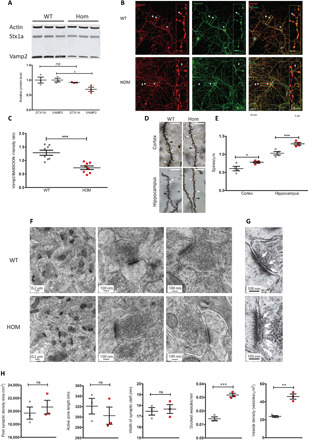
Molecular and cellular deficits in *Vamp2^rlss^*. (**A**) Western blots of whole-brain lysates in WT (gray) and homozygotes (*Vamp2^rlss^*, red). STX1a and VAMP2 are quantitated relative to actin levels. (**B**) WT and *Vamp2^rlss^* hippocampal cell cultures immunostained with VAMP2 (red) and BASSOON (green); colocalization is shown in merged channel images (yellow, right panels). Arrows indicate presynaptic active zone containing both VAMP2 and BASSOON. Insert shows a close up of stained neuron showing VAMP2, BASSOON, and colocalization. Scale bars as shown. (**C**) VAMP2 expression in DIV15 primary cell culture. VAMP2 fluorescence intensity was normalized to that of BASSOON. (**D**) Golgi-stained sections showing mushroom (white arrowhead), long (black arrowhead), and stubby (white arrow) spines. Scale bar, 10 μm. (**E**) Spine counts per unit length in WT (gray) and Vamp2rlss (red) samples. (**F**) Electron micrographs of hippocampal sections. Scale bars as shown. (**G**) Close-up of images in (F) showing docked vesicles (arrows). Scale bars as shown. (**H**) Measurement of synaptic parameters in WT (gray) and *Vamp2^rlss^* (red) mice. Individual data points are shown as is mean ± SEM, *P < 0.05, **P < 0.01, ***P < 0.001.

Investigations into the nature of the *Vamp2^rlss^* mutation were driven by observations that this allele was phenotypically distinct from either heterozygous or homozygous null mutant mice (*11*). Western blots from whole brain, cortex, or hippocampus indicated that the mutant protein was not as stable as WT, with levels ranging from 25 to 65% of controls. Notably, the associated tSNARE protein syntaxin 1a (STX1A) was unaffected (Fig. 4A and fig. S3A). Immunofluorescence labeling of hippocampal neuronal cultures confirmed that synaptic VAMP2 levels, when normalized to that of the synaptic active zone marker BASSOON, were about 50% that of controls (Fig. 4, B and C, and fig. S4, C to E), and these deficits were mirrored in synaptic fractions from cortex or hippocampus (fig. S3, B and C). We investigated whether residual mutant VAMP2 protein was functionally anomalous. On the basis of evidence that mutations in the transmembrane domain of VAMP2 may affect vesicle fusion, fusion pore dynamics, and exocytosis (*12*–*14*), we conducted reciprocal immunoprecipitations (IPs) using antibodies to VAMP2 or STX1A. Using either native protein or tagged expression protein for IP, we were unable to detect a consistent difference in protein interaction (fig. S3, D to F), although neither may reflect the true dynamism of the interaction between vSNARE and tSNARE components in the context of their physiological lipid environment. Elsewhere, we detected what may be a compensatory homeostatic response in mutants. In cultured neurons, dendritic branching and synaptic density were higher in mutants (fig. S4, A and B). Golgi-stained brain sections also revealed an increase in dendritic spine count (Fig. 4, D and E) with greater numbers of immature spines in mutants (fig. S5). At an ultrastructural level, no gross differences in hippocampal synaptic measures were identified. However, the density of synaptic vesicles and the density of docked vesicles were ~2-fold greater in homozygotes (Fig. 4, F to H).

### Substantial functional vesicular deficits are detectable in *Vamp2^rlss^* hippocampal cultures and slices

**Fig. 5:**
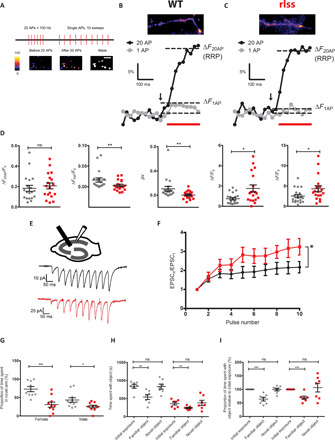
Electrophysiological and behavioral anomalies in *Vamp2^rlss^*. (**A**) Relative RRP size was determined by measuring the change in fluorescence associated with 20 AP stimuli. This was followed by measuring responses to single AP stimulation. Average traces of sypHy responses from 20 to 40 boutons to a 20 AP × 100 Hz burst (∆*F*_20AP_ black, beginning of stimulation indicated by arrow) and a single AP (∆*F*_1AP_ gray, average of 10 sweeps) in WT (**B**) and mutant (**C**) neurons. Average release probability of individual RRP vesicles was estimated in each experiment as *pv* = ∆*F*_1AP_/∆*F*_20AP_. (**D**) Comparison of average RRP size, single AP responses, *pv*, relative total recycling pool of vesicles, and the total number of SVs in WT (gray) and *Vamp2^rlss^* (red) neurons. Note marked reduction in *pv* in response to a single AP in mutant neurons. *n* = 19 coverslips for WT and *n* = 18 coverslips for Hom from *n* = 5 independent cultures. (**E**) Experimental schematics and representative excitatory postsynaptic current (EPSC) responses to 10 AP × 20 Hz stimulation in WT (black) and *Vamp2^rlss^* (red) CA1 pyramidal cells. (**F**) Comparison of short-term synaptic facilitation. WT *n* = 8, mutant *n* = 7. (**G**) Percentage time spent in the novel arm of a *Y* maze. (**H**) Absolute times spent with object in novel object task. (**I**) Proportion of time spent with object relative to time spent with original object in novel object task. WT shown in grey; rlss shown in red. Individual data points are shown as is mean ± SEM, *P < 0.05, **P < 0.01, ***P < 0.001.

We tested the effect of *Vamp2^rlss^* on synaptic vesicle release and functional vesicular pool sizes in hippocampal synapses in neuronal cultures. Cultured neurons were transduced with the fluorescence vesicular release reporter sypHy (*15*). Active synaptic boutons were identified using a short burst of high-frequency stimulation (20 action potentials, APs × 100 Hz), which triggers exocytosis of the readily releasable pool (RRP) of vesicles. Fluorescence responses to single APs were next measured in identified presynaptic boutons (Fig. 5, A and B), enabling an estimate of the average release probability (*pv*) of individual RRP vesicles (*16*). While functional RRP size was similar, *pv* in response to a single AP was profoundly decreased in *Vamp2^rlss^* neurons (Fig. 5C). Furthermore, in agreement with electron microscopy data, we observed a ~2-fold increase in the total recycling pool (TRP) size and the total number of synaptic vesicles (SV) in *Vamp2^rlss^* neurons as measured by fluorescence sypHy responses to high K+- and NH_4_Cl-containing solutions, respectively (Fig. 5D). Decreases in *pv* are normally associated with an increase in short-term synaptic facilitation (*17*). Experiments in acute hippocampal slices revealed a ~2-fold increase in short-term facilitation in *Vamp2^rlss^* slices during short high-frequency stimulation of the Shaffer collateral synapses (Fig. 5, E and F).

Given the role of VAMP2 in fundamental neural mechanisms and the electrophysiological deficits seen in homozygotes, we expected mutants to display behavioral and sensory discrepancies in addition to changes in sleep. Unexpectedly, performance in a wide test battery indicated only mild behavioral anomalies (fig. S6), while sensory function was unchanged or even improved (figs. S7 and S8). However, homozygous behavior in a number of tests pointed to deficits in working memory (*Y* maze and novel object recognition), attention (marble burying, novel object recognition), and social instability (social dominance tube test) (Fig. 5, G to I). These irregularities were defined further through display of numerous stereotypical behaviors analyzed using home-cage continuous video monitoring (movie S1).

## DISCUSSION

The sleep deficit identified here in *Vamp2^rlss^* mice uncovers a previously unrecognized role for VAMP2 in sleep and may indicate a critical function for the transmembrane domain in which the mutation occurs. VAMP2 function has been profoundly well characterized with respect to its fundamental role in vesicle fusion and exocytosis (*18*). Nevertheless, its functional characterization in mammals in vivo has been hampered as the *Vamp2^−/−^* mouse mutant is perinatal lethal, while *Vamp2^+/−^* shows no behavioral phenotype beyond mild improvements in rotarod performance (*19*). The recent identification of multiple de novo variants of *VAMP2* in humans has highlighted how mutations affecting the SNARE zippering mechanism can have severe developmental consequences (*20*). In contrast, the relatively mild consequences of the *Vamp2^rlss^* mutation in mice has enabled us to examine features of VAMP2 function in adults. Moreover, recent studies (*21*, *22*) identified *VAMP2* lead SNP associations for chronotype and sleep duration measures, suggesting that subtle variations in VAMP2 function could influence sleep quality in humans.

*Vamp2^rlss^* affords a new model to study the genetic control of vigilance state switching, especially with respect to the role of underlying local and global network mechanisms. We demonstrate that *Vamp2^rlss^* mice display an increased stability of both wakefulness and sleep, suggesting that impaired vesicular release results in an increased state inertia that may be related to switches from low-pass to high-pass filtering properties of synapses (*17*). It is likely that this effect is mediated by changes within specific local brain regions directly involved in sleep-wake control (*23*–*25*). The traditional view is that the occurrence of a specific state of vigilance is regulated by a distributed subcortical network consisting of mutually inhibiting sleep- and wake-promoting neurons (*26*–*28*). However, how precisely the dynamic balance among the circuitries involved in sleep-wake control is maintained is yet unclear. We surmise that a globally decreased synaptic efficiency in *Vamp2^rlss^* mice leads to a functional weakening of those synaptic connections that mediate rapid state switching and, therefore, increases “resistance” of neuronal populations, promoting a specific state of vigilance, to inputs arising from competing areas (*26*, *29*). For example, reduced efficacy of inhibitory pathways between the sleep- and wake-promoting regions is expected to prevent an early termination of sleep and wake episodes and thus result in increased state stability. Therefore, we argue that, in physiological conditions, global shifts in network excitability arising from changes in fundamental neuronal properties, such as synaptic vesicular release, may facilitate processes regulated at the local level, such as sleep-wake control.

## MATERIALS AND METHODS

### Animals

Animal studies were performed under guidance from the Medical Research Council in Responsibility in the Use of Animals for Medical Research (July 1993), the University of Oxford’s Policy on the Use of Animals in Scientific Research, and Home Office Project Licenses 30/2686 and 80/2310. When not tested, mice were housed in individually ventilated cages under 12/12-hour light/dark conditions with food and water available ad libitum. Inbred strains and mutant colonies were maintained at MRC Harwell, and cohorts were shipped as required. ENU mutagenesis and animal breeding regimes were performed as previously reported (*8*). Phenotyping was performed on mouse cohorts that were partially or completely congenic on the C57BL/6J or C3H.Pde6b+ (*30*) background.

### Video tracking screen for immobility-defined sleep

Video tracking was performed as described (*7*). Briefly, mice were singly housed and placed in light-controlled chambers with charge-coupled device cameras positioned above the cages (Maplin, UK). Monitoring during the dark was performed using infrared illumination. Analysis of videos by ANYmaze software (Stoelting) was used to track mouse mobility and immobility-defined sleep (successive periods of >40-s immobility). Mice for screening were provided by a large-scale ENU mutagenesis project (*8*).

### Gene mapping and sequencing

Affected individuals from G3 pedigrees were used for mapping and mutation detection. Mutations were mapped using the Illumina GoldenGate Mouse Medium Density Linkage Panel (Gen-Probe Life Sciences Ltd., UK), providing a map position resolution of about ~20 Mb. Following mapping, whole-genome sequencing of the G1 founder male was carried out as described (*8*); variants were confirmed by Sanger sequencing.

### EEG surgery, recording, and analysis

Average ages and weights of mice at the time of surgery were 23.8 ± 1.2 weeks and 31.9 ± 0.8 g, and 14.7 ± 1.5 weeks and 31.7 ± 1.5 g for *Vamp2^rlss^* and WT mice, respectively. Age differences were permitted so that animals were matched by weight. Previous data indicate that this age difference does not contribute to the differences in sleep observed (*31*, *32*). *Vamp2^rlss^* mice were fed a high-nutrient diet in addition to regular pellets. Surgical methods, including drug administration and aseptic techniques, were as described (*32*). EEG screws (Fine Science Tools Inc.) were placed in the frontal (anteroposterior, +2 mm; mediolateral, 2 mm) and occipital (anteroposterior, −3.5 to −4 mm; mediolateral, 2.5 mm) cortical regions; reference and ground screws were implanted above the cerebellum and contralaterally to the occipital screw, respectively. Two stainless steel wires were inserted in the neck muscle for EMG. Mice were singly housed postsurgery.

After 2 weeks of recovery, mice were placed in cages (20.3 × 32 × 35 cm^3^) in ventilated, sound-attenuated Faraday chambers (Campden Instruments, UK) under a 12:12-h light-dark cycle with food and water available ad libitum. EEG and EMG data were filtered (0.1 to 100 Hz), amplified (PZ5 NeuroDigitizer preamplifier, Tucker-Davis Technologies), and stored on a local computer (sampling rate, 256.9 Hz). Signals were extracted and converted using custom-written MATLAB (The MathWorks Inc., USA) scripts and the open-source Neurotraces software. Vigilance states—wake, NREM, REM, or brief awakenings (short arousals ≤16 s during sleep)—were scored manually using the SleepSign software (Kissei Comtec Co., Nagano, Japan). EEG power density spectra were computed by a fast Fourier transform (Hanning window; 0.25-Hz resolution). More detailed recording and initial analysis methods were as published (*32*). Unless otherwise stated, NREM and wake episodes were defined as episodes of at least 1-min duration, allowing brief interruptions ≤16 s (e.g., brief awakenings or brief REM attempts within NREM). REM sleep episodes could be as short as 4 s and bear interruptions ≤4 s.

### Behavioral phenotyping

#### Circadian wheel running

Circadian wheel running was performed as previously reported (*33*). Briefly, mice were singly housed in cages containing running wheels, placed in light-controlled chambers, and wheel running activity was monitored via ClockLab (Actimetrics). Animals were monitored for 5 days in a 12-hour light/dark cycle followed by 12 days in constant darkness.

#### Passive infrared analysis

Mice were additionally analyzed for circadian activity using the COMPASS passive infrared system as described (*9*). Animals were monitored for 4 days in a 12-hour light/dark cycle followed by 10 days in constant darkness.

#### Open-field behavior

Mice were placed into one corner of a walled arena (45 cm by 45 cm) and allowed to explore on two consecutive days for 30 min (*34*). Animal movements and position were tracked using EthoVision XT analysis software (Noldus).

#### Light/dark box

Individual mice were placed into one corner of an enclosed arena separated into light and dark compartments (*35*). Over 20 min, animal movements and positions were monitored by EthoVision XT.

#### Marble burying

Briefly, a cage was prepared with approximately 5-cm-deep sawdust bedding (*35*). Nine marbles were placed on the surface of the sawdust, evenly spaced in a regular pattern. The mouse was introduced and left in the cage with the marbles for 30 min. After 30 min, the number of marbles remaining unburied, partially buried, or completely buried was counted. Statistical differences were determined using the Mann-Whitney test.

#### Acoustic startle response and prepulse inhibition

Acoustic startle response and prepulse inhibition were measured as in (*36*). Mice were placed in the apparatus (Med Associates, VT, USA), and responses to sound stimuli were measured via an accelerometer.

#### Novel object recognition

A modified version of the novel object recognition task adopted for use in the home cage was used using video analysis of behaviors. On day 1, animals were presented with one novel object (glass jar or LEGO bars) in a corner of their home cage for 30 min at the beginning of the dark phase. On the following night, the same object was introduced at the same position in the home cage, again at the beginning of the dark phase for 30 min. On the third night, a second object, different from the first one, was introduced at a different position in the home cage but at the same time of night and for the same duration. Time spent inspecting objects was measured manually from video recordings using a stop watch.

#### Social dominance test

Dominant and submissive behaviors were assessed for pairs of male mice using a specialized Plexiglass tube (*35*). Pairs of mice, one WT and one homozygous mutant from different group-housed cages, were placed in the tube, and behavior was registered as dominant (“win”) or subordinate. Five pairs were used, and total numbers of “wins” per genotype was recorded. Statistical differences were ascertained using a chi-square test.

#### Y maze

A forced alternation *Y* maze test was used to evaluate short-term working memory in mice (*35*). Mice were video tracked at all times using EthoVision XT; preference for the novel arm is indicated by an occupancy of greater than 33%.

#### Mechanical sensitivity, von Frey test

Mechanical sensitivity was assessed as in (*35*) by determining the withdrawal threshold of the hind limb to a mechanical stimulation applied under the foot pad using an electronic von Frey apparatus (MouseMet, TopCat Metrology, UK). Five consecutive measurements per hind paw were taken, and the average of the last four was calculated.

#### Heat sensitivity, hot plate test

A hot plate (BioSeb, Chaville, France) set at 51°C was used for this test (*35*). Animals were placed on the plate, and the latency to the first paw reflex (withdrawal reflex of one paw) was measured.

#### Optokinetic response

Visual acuity was assessed by head tracking response to a virtual reality optokinetic system (Cerebral Mechanics Inc.) (*37*).

#### Auditory brainstem response

Auditory brainstem response (ABR) tests were performed using a click stimulus in addition to frequency-specific tone-burst stimuli as described (*38*). ABRs were collected, amplified, and averaged using TDT System 3 (Tucker Davies Technology) driven by BioSig RZ (v5.7.1) software. All stimuli were presented free field to the right ear of the anesthetized mouse, starting at 70-dB sound pressure level (SPL) and decreasing in 5-dB steps. Auditory thresholds were defined as the lowest dB SPL that produced a reproducible ABR trace pattern and were determined visually.

#### Grip strength

Grip strength was assessed using a Grip Strength Meter (BioSeb, Chaville, France). Readings were taken from all four paws, three times per mouse as per the manufacturer’s instructions (*34*). Measures were averaged and normalized to body weight.

#### Home-cage analysis

Group-housed animals were monitored as described (*39*). Briefly, group-housed mice were tagged with radio-frequency identification (RFID) microchips at 9 weeks of age and placed in the home-cage analysis system (Actual Analytics, Edinburgh), which captured mouse behavior using both video tracking and location tracking using RFID coordinates.

### Golgi-Cox staining, spine count analysis

Brains dissected from 16-week-old females were used for analysis. Golgi-Cox neuronal staining was performed using the FD Rapid GolgiStain Kit (FD NeuroTechnologies Inc., USA) according to the manufacturer’s instructions. One hundred–micrometer sections were taken using a vibratome, mounted upon charged slides, cleared in Histo-Clear (National Diagnostics, UK), and coverslipped. Neurons were viewed on an AxioObserver Z1 (Zeiss) microscope. Z-stack images were processed using extended depth of focus and Zen software (Zeiss). Visualization and measurements were taken using ImageJ (http://rsbweb.nih.gov/ij/). The number and type of spines on each neurite—stubby, long, mushroom, and branched (*40*)—were counted. At least 50 neurites per region per animal were analyzed.

### Electron microscopy

Brains were fixed by cardiac perfusion [buffer solution: sodium cacodylate buffer (pH 7.2) containing 4.35% sucrose, fixation solution: 2.5% glutaraldehyde and 4% paraformaldehyde in buffer solution], and then removed and coronally cut into 350-μm-thick sections using a Vibratome (Leica VT1000 S, Leica Biosystems, Nußloch, Germany). Selected regions from hippocampus were dissected, further fixed with osmium tetroxide and uranyl acetate, and dehydrated. After embedding in epoxy resin, the tissue was cut into 70-nm-thick sections and poststained with uranyl acetate and lead citrate for imaging using a transmission electron microscope FEI Tecnai 12 TEM (FEI, Hillsboro, OR, USA). To avoid any bias between WT and homozygous synapses, overview pictures were taken and the number of visible synapses in this plane was counted by hand. Higher-magnification pictures were exported into ImageJ, and measurements were taken for each synapse. At least 60 synapses were analyzed per animal.

### Plasmids

Plasmid constructs were generated using Invitrogen Gateway gene cloning technology. WT STX1a and VAMP2 coding sequences were first amplified from complementary DNA (cDNA) libraries with proofreading polymerase (Platinum SuperFi DNA Polymerase, Invitrogen). Mutant VAMP2 (mVAMP2) was amplified using cDNA obtained from mouse brain. Primers for cloning were as follows: STX1a, CACCAAGGACCGAACCCAGGA (forward) and CTATCCAAAGATGCCCCCG (reverse); VAMP2, CACCTCGGCTACCGCTGCCA (forward) and TTAAGTGCTGAAGTAAACGATGA (reverse). Amplified DNA fragments were cloned into pENTR/D-TOPO vector. STX1a was subsequently transferred to pcDNA 3.1/nV5-DEST Mammalian Expression Vector (V5 Epitope, Invitrogen), and both WT and mutant VAMP2 sequences were shuttled into Gateway pDEST 26 Vector (6xHis tag, Invitrogen). The in-frame V5-STX1a and 6xHis-(m)VAMP2 sequences were verified by DNA sequencing.

### Antibodies

Primary antibodies for IP and Western blotting included anti-V5 and anti-His (R&D systems), mouse anti-VAMP2 (SySy Synaptic Systems, Göttingen, Germany), and rabbit anti-syntaxin (Sigma, St. Louis, Missouri, USA). Secondary antibodies were IRDye 800CW goat anti-mouse immunoglobulin G (IgG) (1:15,000) and IRDye 680RD goat anti-rabbit IgG (1:15,000). Primary antibodies for immunofluorescence studies were rabbit anti-VAMP2, guinea-pig anti-BASSOON, and mouse anti-MAP2 (Synaptic Systems 188004, Novus SAP7F407, and Synaptic Systems 188004, respectively). The corresponding secondary antibodies included 488 donkey anti-mouse antibody (Invitrogen), 596 goat anti–guinea-pig antibody (Invitrogen), and 647 goat anti-rabbit antibody (Life Technologies).

### Cell culture and transfection

Human embryonic kidney 293T (HEK293T) cells were maintained in T75 flasks containing Dulbecco’s modified Eagle’s medium (Gibco) supplemented with 10% fetal bovine serum (Gibco) and penicillin-streptomycin (Gibco). Before transfection, cells were seeded at 2 × 10^5^ cells per well in a six-well plate and left to adhere overnight. Plasmids (total of 2.5 μg) were transfected with Lipofectamine 2000 (Invitrogen) at a ratio of 1:2 in Opti-MEM I Reduced Serum Medium (Gibco). Cotransfections of V5-STX1a and 6xHis-mVAMP2 were adjusted so that equivalent amounts of the two proteins were expressed in culture. Transfected cells were then incubated at 37°C for 16 to 24 hours to express tagged proteins.

### Cell and tissue lysis and fractionation

Cells were harvested 24 hours after transfection and lysed by suspension in ice-cold radioimmunoprecipitation assay buffer (Sigma-Aldrich, R0278-50ML) supplemented with protease inhibitor mixture (Roche Diagnostics). Suspensions were incubated at 4°C for 30 min and centrifuged at 4°C, 14,000 rpm for 20 min. Tissue lysis and crude synaptic fractionation were performed using Syn-PER Synaptic Protein Extraction Reagent (Thermo Fisher Scientific) following the manufacturer’s instructions.

### Immunoprecipitation

For IP of tagged proteins, the Thermo Scientific Pierce Crosslink IP Kit (Thermo Fisher Scientific, 26147) was used as per the manufacturer’s instructions. Briefly, antibodies were cross-linked to Pierce Protein A/G Plus Agarose to avoid antibody contamination at later steps. Total cell extracts containing V5-STX1a and 6xHis-(m)VAMP2 were precleared with Pierce Control Agarose resin in Pierce Spin Columns for 30 min at 4°C and then spun to collect the flow-through, discarding the control agarose columns. Precleared samples were then added to antibody cross-linked columns and incubated at 4°C overnight. After incubation, columns were spun and the flow-through was discarded. The columns were then washed with TBST [25 mM tris-HCl (pH7.4), 138 mM NaCl, 0.05% Tween 20, National Diagnostics, EC-882] and conditioning buffer to remove nonbound components. The tagged protein complexes were then recovered with the elution buffer supplied. IP of native protein complexes from hippocampal lysates was carried out as described previously (*36*).

### Electrophoresis and Western blotting

Samples were prepared in NuPAGE LDS Sample Buffer (Invitrogen, NP0007) supplemented with NuPAGE Sample Reducing Agent (Invitrogen, NP0004) and heated at 70°C for 10 min. Equal amounts of protein were loaded and resolved on NuPAGE Bis-Tris 4 to 12% gel (Invitrogen, NP032A) and then transferred to nitrocellulose membrane contained in iBlot Gel Transfer Stacks (Invitrogen, NM040319-01) using an iBlot Gel Horizontal Transfer Device (Invitrogen, IB21001). After blotting, membranes were incubated with primary antibodies, diluted in TBST containing 5% skimmed milk, at 4°C overnight. Subsequently, membranes were washed with TBST and incubated with IR700 and IR800 secondary antibodies (LI-COR Biosciences, Lincoln, NE) for 2 hours at room temperature. After further washes, immunoreactive bands were visualized using an Odyssey Infrared Imaging System (LI-COR Biosciences, Lincoln, NE).

### Hippocampal culture and transduction

Hippocampal neuronal cultures were prepared from P0 to P1 littermate WT or *Vamp2^rlss^* homozygous pups (*41*), Neurons were plated on poly-l-lysine (1 mg/ml; Sigma-Aldrich)–treated 19-mm glass coverslips at a density of 80,000 to 120,000 per coverslip. Immunofluorescence and sypHy imaging experiments were performed between 15 and 23 days in vitro (DIV). At DIV seven, neurons were transduced with pFU_sypHy lentivirus containing syntophysin-pHluorin cDNA under control of a ubiquitin promoter. The pFU_sypHy plasmid was provided by A. Maximov (The Scripps Research Institute, La Jolla, USA).

### Immunofluorescence labeling

To evaluate VAMP2 expression and localization, primary hippocampal neurons were fixed and stained after DIV 15 to 23. Hippocampal cultures were first washed in phosphate-buffered saline, then fixed with 4% paraformaldehyde (PFA) for 10 min, and blocked in a 20% donkey serum blocking solution in TBST for 1 hour at room temperature. All primary antibody incubations were conducted over two nights at 4°C. Secondary antibody incubations were for 2 hours at room temperature. All antibodies were applied in 1% blocking serum to minimize antibodies binding to the plasticware. Samples were washed three times with TBST for 10 min at room temperature between steps. After antibody treatment and final washes, coverslips with cells were mounted onto glass slides with mounting medium (ProLong, Gold Antifade Reagent, Invitrogen) and then dried in the dark overnight at 4°C before imaging.

### Image and Sholl analysis

Fluorescence images were acquired at room temperature with an inverted confocal microscope (Zeiss, LMS 710) using a Plan-Apochromat 40×/1.4 (oil, DIC, M27) objective or 20×/0.8 (M27) objective to get four-layer Z-stacks. For the synaptic intensity experiment, the same settings for laser power, photomultiplier tube (PMT) gain and offset, and Z-stack thickness were used. The pinhole size was set to 1 Airy unit for the shortest wavelength channel and the faintest image. The Z-stacks acquired were compressed into single-layer images by maximum projection. For intensity quantification, multichannel fluorescence images were first converted into monochromatic and color-inverted pictures by ImageJ. All fluorescence intensity analysis was conducted at the same setting.

For synaptic distribution analysis, conditions were optimized independently to capture data from all neurites for a single cell. PMT gain was optimized individually, using 10% area oversaturation on the shortest wavelength as a reference for all channels (*n* > 12 for each genotype). Then, Z-stacks acquired were compressed into single-layer images by maximum projection. To quantify synaptic protein distribution further, we investigated VAMP2 distribution pattern by analyzing VAMP2-containing synaptic projections and associated neuritic branching using a MATLAB-based SynD program (*42*). Multichannel fluorescence images were acquired using ZEN software and converted into monochromatic and color-inverted pictures by ImageJ for further analyses. Statistical comparisons were performed using a Mann-Whitney test with genotype and intensity as independent factors.

### SypHy fluorescence imaging experiments in neuronal cultures

Primary hippocampal neurons were maintained in modified Tyrode solution containing 125 mM NaCl, 2.5 mM KCl, 2 mM MgCl_2_, 2 mM CaCl_2_, 30 mM glucose, and 25 mM Hepes (pH 7.4) supplemented with 2,3-dioxo-6-nitro-7-sulfamoyl-benzo[*f*]quinoxaline (NBQX) (10 μM; Ascent Scientific) and DL-AP5 (50 μM). APs were evoked by field stimulation via platinum bath electrodes separated by 1 cm (12.5 to 15 V, 1-ms pulses). To estimate the relative TRP size and total numbers of SVs, neurons were perfused with Tyrode modified solution containing either 45 mM KCl or 50 mM NH_4_CL, respectively. Images were acquired via 63× objective using a Prime 95B CMOS camera (Photometrics) mounted on an inverted Ziess Axiovert 200 microscope equipped with a 488-nm excitation light-emitting diode light source and a 510 long-pass emission filter. Exposure time was 25 ms.

### Image and data analysis of sypHy experiments

Images were analyzed in ImageJ and MATLAB using custom-written plugins. A binary mask was placed on all varicosities that were stably in focus throughout all trials and responded to 20 AP 100-Hz burst stimulation. To estimate sypHy fluorescence changes induced by 20 AP × 100 Hz, 1 AP, KCl, and NH_4_CL, the difference between the mean of eight frames before and eight frames after the stimulus was calculated for all synapses in the field of view (10 to 100 range). After subtracting the background, the data were normalized to the resting sypHy signal (*F*_0_).

### Acute slice preparation and electrophysiological recordings

Whole-cell electrophysiological recordings of excitatory postsynaptic currents (EPSCs) in Shaffer collaterals in acute hippocampal slices from 1- to 2-month-old mice have been performed as previously described (*43*). The extracellular perfusion solution contained 119 mM NaCl, 2.5 mM KCl, 2.5 mM CaCl_2_, 1.3 mM MgSO_4_, 1.25 mM NaH_2_PO_4_, 25 mM NaHCO_3_, and 10 mM glucose. NMDA (*N*-methyl-d-aspartate) and GABA_A_ (γ-aminobutyric acid type A) receptors were blocked routinely with 50 μM D-aminophosphonovalerate (D-APV) and 100 μM picrotoxin. A concentric bipolar stimulating electrode (FHC), connected to a constant current stimulator, was placed in the stratum radiatum, and 10 pulses were elicited at 20 Hz. Stimulation intensity ranged from 20 to 320 μA and was adjusted to obtain the first EPSC amplitude in the range of 25 to 100 pA. The elicited EPSCs were recorded from pyramidal neurons of CA1 that were whole-cell patch clamped using 4- to 6-megohm resistance recording pipettes and held at −70 mV. The pipette solution contained 125 mM Cs-gluconate, 10 mM Hepes, 10 mM Na-phosphocreatine, 8 mM NaCl, 4 mM Mg-ATP, 0.3 mM Na_3_-GTP, 0.2 mM EGTA, 5 mM TEA-Cl, 0.5 mM biocytin. Data were acquired using a PCI-6221 interface (National Instruments) and custom software (LabVIEW). Currents were low-pass filtered (4 to 5 kHz) and digitized at 10 to 20 kHz before analysis.

### Data analysis and statistics

Unless otherwise stated, statistical differences were established using a Student’s *t* test. Behavioral phenotyping, synaptic spine analysis, electron microscopy, Western blot quantification, and immunohistochemical quantification were analyzed using GraphPad Prism 7 (GraphPad Software). EEG sleep analysis was performed using MATLAB (MathWorks) and SPSS (IBM Corp). SypHy fluorescence imaging experiments were analyzed using GraphPad Prism 7 and SigmaPlot (Systat Software Inc.). The electrophysiological recordings were analyzed using LabVIEW (National Instruments), PClamp 10 (Molecular Devices), and MATLAB (MathWorks) software. Significance level for all analysis was set at *P* < 0.05.

## Supplementary Material

abb3567_Table_S1.xlsx

abb3567_SM.pdf

abb3567_Movie_S1.mp4

## SUPPLEMENTARY MATERIALS

Supplementary material for this article is available at http://advances.sciencemag.org/cgi/content/full/6/33/eabb3567/DC1

## Appendix

View/request a protocol for this paper from *Bio-protocol*.

## References

[1] Gent T. C., Bassetti C. L. A., Adamantidis A. R., Sleep-wake control and the thalamus. Curr. Opin. Neurobiol. 52, 188–197 (2018). 30144746 10.1016/j.conb.2018.08.002

[2] Saper C. B., Fuller P. M., Wake-sleep circuitry: An overview. Curr. Opin. Neurobiol. 44, 186–192 (2017). 28577468 10.1016/j.conb.2017.03.021 PMC5531075

[3] Godinho S. I. H., Maywood E. S., Shaw L., Tucci V., Barnard A. R., Busino L., Pagano M., Kendall R., Quwailid M. M., Romero M. R., O’neill J., Chesham J. E., Brooker D., Lalanne Z., Hastings M. H., Nolan P. M., The after-hours mutant reveals a role for Fbxl3 in determining mammalian circadian period. Science 316, 897–900 (2007). 17463252 10.1126/science.1141138

[4] Parsons M. J., Brancaccio M., Sethi S., Maywood E. S., Satija R., Edwards J. K., Jagannath A., Couch Y., Finelli M. J., Smyllie N. J., Esapa C., Butler R., Barnard A. R., Chesham J. E., Saito S., Joynson G., Wells S., Foster R. G., Oliver P. L., Simon M. M., Mallon A.-M., Hastings M. H., Nolan P. M., The regulatory factor ZFHX3 modifies circadian function in SCN via an AT motif-driven axis. Cell 162, 607–621 (2015). 26232227 10.1016/j.cell.2015.06.060 PMC4537516

[5] Vitaterna M. H., King D. P., Chang A. M., Kornhauser J. M., Lowrey P. L., McDonald J. D., Dove W. F., Pinto L. H., Turek F. W., Takahashi J. S., Mutagenesis and mapping of a mouse gene, Clock, essential for circadian behavior. Science 264, 719–725 (1994). 8171325 10.1126/science.8171325 PMC3839659

[6] Funato H., Miyoshi C., Fujiyama T., Kanda T., Sato M., Wang Z., Ma J., Nakane S., Tomita J., Ikkyu A., Kakizaki M., Hotta-Hirashima N., Kanno S., Komiya H., Asano F., Honda T., Kim S. J., Harano K., Muramoto H., Yonezawa T., Mizuno S., Miyazaki S., Connor L., Kumar V., Miura I., Suzuki T., Watanabe A., Abe M., Sugiyama F., Takahashi S., Sakimura K., Hayashi Y., Liu Q., Kume K., Wakana S., Takahashi J. S., Yanagisawa M., Forward-genetics analysis of sleep in randomly mutagenized mice. Nature 539, 378–383 (2016). 27806374 10.1038/nature20142 PMC6076225

[7] Fisher S. P., Godinho S. I. H., Pothecary C. A., Hankins M. W., Foster R. G., Peirson S. N., Rapid assessment of sleep-wake behavior in mice. J. Biol. Rhythms 27, 48–58 (2012). 22306973 10.1177/0748730411431550 PMC4650254

[8] Potter P. K., Bowl M. R., Jeyarajan P., Wisby L., Blease A., Goldsworthy M. E., Simon M. M., Greenaway S., Michel V., Barnard A., Aguilar C., Agnew T., Banks G., Blake A., Chessum L., Dorning J., Falcone S., Goosey L., Harris S., Haynes A., Heise I., Hillier R., Hough T., Hoslin A., Hutchison M., King R., Kumar S., Lad H. V., Law G., MacLaren R. E., Morse S., Nicol T., Parker A., Pickford K., Sethi S., Starbuck B., Stelma F., Cheeseman M., Cross S. H., Foster R. G., Jackson I. J., Peirson S. N., Thakker R. V., Vincent T., Scudamore C., Wells S., El-Amraoui A., Petit C., Acevedo-Arozena A., Nolan P. M., Cox R., Mallon A.-M., Brown S. D. M., Novel gene function revealed by mouse mutagenesis screens for models of age-related disease. Nat. Commun. 7, 12444 (2016). 27534441 10.1038/ncomms12444 PMC4992138

[9] Brown L. A., Hasan S., Foster R. G., Peirson S. N., COMPASS: Continuous open mouse phenotyping of activity and sleep status. Wellcome Open Res. 1, 2 (2016). 27976750 10.12688/wellcomeopenres.9892.2 PMC5140024

[10] Ang G., McKillop L. E., Purple R., Blanco-Duque C., Peirson S. N., Foster R. G., Harrison P. J., Sprengel R., Davies K. E., Oliver P. L., Bannerman D. M., Vyazovskiy V. V., Absent sleep EEG spindle activity in GluA1 (Gria1) knockout mice: Relevance to neuropsychiatric disorders. Transl. Psychiatry 8, 154 (2018). 30108203 10.1038/s41398-018-0199-2 PMC6092338

[11] Schoch S., Deák F., Königstorfer A., Mozhayeva M., Sara Y., Südhof T. C., Kavalali E. T., SNARE function analyzed in synaptobrevin/VAMP knockout mice. Science 294, 1117–1122 (2001). 11691998 10.1126/science.1064335

[12] Chiang C.-W., Chang C.-W., Jackson M. B., The transmembrane domain of synaptobrevin influences neurotransmitter flux through synaptic fusion pores. J. Neurosci. 38, 7179–7191 (2018). 30012692 10.1523/JNEUROSCI.0721-18.2018 PMC6083459

[13] Dhara M., Yarzagaray A., Makke M., Schindeldecker B., Schwarz Y., Shaaban A., Sharma S., Böckmann R. A., Lindau M., Mohrmann R., Bruns D., v-SNARE transmembrane domains function as catalysts for vesicle fusion. eLife 5, e17571 (2016). 27343350 10.7554/eLife.17571 PMC4972536

[14] Hastoy B., Scotti P. A., Milochau A., Fezoua-Boubegtiten Z., Rodas J., Megret R., Desbat B., Laguerre M., Castano S., Perrais D., Rorsman P., Oda R., Lang J., A central small amino acid in the VAMP2 transmembrane domain regulates the fusion pore in exocytosis. Sci. Rep. 7, 2835 (2017). 28588281 10.1038/s41598-017-03013-3 PMC5460238

[15] Granseth B., Odermatt B., Royle S. J., Lagnado L., Clathrin-mediated endocytosis is the dominant mechanism of vesicle retrieval at hippocampal synapses. Neuron 51, 773–786 (2006). 16982422 10.1016/j.neuron.2006.08.029

[16] Ariel P., Ryan T. A., Optical mapping of release properties in synapses. Front Neural Circuits 4, 18 (2010). 20802854 10.3389/fncir.2010.00018 PMC2928663

[17] Abbott L. F., Regehr W. G., Synaptic computation. Nature 431, 796–803 (2004). 15483601 10.1038/nature03010

[18] Han J., Pluhackova K., Böckmann R. A., The multifaceted role of SNARE proteins in membrane fusion. Front. Physiol. 8, 5 (2017). 28163686 10.3389/fphys.2017.00005 PMC5247469

[19] Monteggia L. M., Lin P.-Y., Adachi M., Kavalali E. T., Behavioral Analysis of SNAP-25 and Synaptobrevin-2 Haploinsufficiency in Mice. Neuroscience 420, 129–135 (2019). 30144509 10.1016/j.neuroscience.2018.08.014 PMC6387657

[20] Salpietro V., Malintan N. T., Llano-Rivas I., Spaeth C. G., Efthymiou S., Striano P., Vandrovcova J., Cutrupi M. C., Chimenz R., David E., Rosa G. D., Marce-Grau A., Raspall-Chaure M., Martin-Hernandez E., Zara F., Minetti C.; Deciphering Developmental Disorders Study; SYNAPS Study Group, Bello O. D., De Zorzi R., Fortuna S., Dauber A., Alkhawaja M., Sultan T., Mankad K., Vitobello A., Thomas Q., Mau-Them F. T., Faivre L., Martinez-Azorin F., Prada C. E., Macaya A., Kullmann D. M., Rothman J. E., Krishnakumar S. S., Houlden H., Mutations in the neuronal vesicular SNARE *VAMP2* affect synaptic membrane fusion and impair human neurodevelopment. Am. J. Hum. Genet. 104, 721–730 (2019). 30929742 10.1016/j.ajhg.2019.02.016 PMC6451933

[21] Dashti H. S., Jones S. E., Wood A. R., Lane J. M., van Hees V. T., Wang H., Rhodes J. A., Song Y., Patel K., Anderson S. G., Beaumont R. N., Bechtold D. A., Bowden J., Cade B. E., Garaulet M., Kyle S. D., Little M. A., Loudon A. S., Luik A. I., Scheer F. A. J. L., Spiegelhalder K., Tyrrell J., Gottlieb D. J., Tiemeier H., Ray D. W., Purcell S. M., Frayling T. M., Redline S., Lawlor D. A., Rutter M. K., Weedon M. N., Saxena R., Genome-wide association study identifies genetic loci for self-reported habitual sleep duration supported by accelerometer-derived estimates. Nat. Commun. 10, 1100 (2019). 30846698 10.1038/s41467-019-08917-4 PMC6405943

[22] Jones S. E., Lane J. M., Wood A. R., van Hees V. T., Tyrrell J., Beaumont R. N., Jeffries A. R., Dashti H. S., Hillsdon M., Ruth K. S., Tuke M. A., Yaghootkar H., Sharp S. A., Jie Y., Thompson W. D., Harrison J. W., Dawes A., Byrne E. M., Tiemeier H., Allebrandt K. V., Bowden J., Ray D. W., Freathy R. M., Murray A., Mazzotti D. R., Gehrman P. R., Lawlor D. A., Frayling T. M., Rutter M. K., Hinds D. A., Saxena R., Weedon M. N., Genome-wide association analyses of chronotype in 697,828 individuals provides insights into circadian rhythms. Nat. Commun. 10, 343 (2019). 30696823 10.1038/s41467-018-08259-7 PMC6351539

[23] Chen K.-S., Xu M., Zhang Z., Chang W.-C., Gaj T., Schaffer D. V., Dan Y., A hypothalamic switch for REM and Non-REM sleep. Neuron 97, 1168–1176.e4 (2018). 29478915 10.1016/j.neuron.2018.02.005

[24] Hayashi Y., Kashiwagi M., Yasuda K., Ando R., Kanuka M., Sakai K., Itohara S., Cells of a common developmental origin regulate REM/non-REM sleep and wakefulness in mice. Science 350, 957–961 (2015). 26494173 10.1126/science.aad1023

[25] Anaclet C., Ferrari L., Arrigoni E., Bass C. E., Saper C. B., Lu J., Fuller P. M., The GABAergic parafacial zone is a medullary slow wave sleep-promoting center. Nat. Neurosci. 17, 1217–1224 (2014). 25129078 10.1038/nn.3789 PMC4214681

[26] Saper C. B., Chou T. C., Scammell T. E., The sleep switch: Hypothalamic control of sleep and wakefulness. Trends Neurosci. 24, 726–731 (2001). 11718878 10.1016/s0166-2236(00)02002-6

[27] Liu D., Dan Y., A motor theory of sleep-wake control: Arousal-action circuit. Annu. Rev. Neurosci. 42, 27–46 (2019). 30699051 10.1146/annurev-neuro-080317-061813

[28] Eban-Rothschild A., Appelbaum L., de Lecea L., Neuronal mechanisms for sleep/wake regulation and modulatory drive. Neuropsychopharmacology 43, 937–952 (2018). 29206811 10.1038/npp.2017.294 PMC5854814

[29] Saper C. B., Fuller P. M., Pedersen N. P., Lu J., Scammell T. E., Sleep state switching. Neuron 68, 1023–1042 (2010). 21172606 10.1016/j.neuron.2010.11.032 PMC3026325

[30] Hart A. W., Kie L. M., Morgan J. E., Gautier P., West K., Jackson I. J., Cross S. H., Genotype-phenotype correlation of mouse *Pde6b* mutations. Invest. Ophthalmol. Vis. Sci. 46, 3443–3450 (2005). 16123450 10.1167/iovs.05-0254

[31] Banks G., Nolan P. M., Peirson S. N., Reciprocal interactions between circadian clocks and aging. Mamm. Genome 27, 332–340 (2016). 27137838 10.1007/s00335-016-9639-6 PMC4935744

[32] McKillop L. E., Fisher S. P., Cui N., Peirson S. N., Foster R. G., Wafford K. A., Vyazovskiy V. V., Effects of aging on cortical neural dynamics and local sleep homeostasis in mice. J. Neurosci. 38, 3911–3928 (2018). 29581380 10.1523/JNEUROSCI.2513-17.2018 PMC5907054

[33] Banks G. T., Nolan P. M., Assessment of circadian and light-entrainable parameters in mice using wheel-running activity. Curr. Protoc. Mouse Biol. 1, 369–381 (2011). 26068996 10.1002/9780470942390.mo110123

[34] Stewart M., Lau P., Banks G., Bains R. S., Castroflorio E., Oliver P. L., Dixon C. L., Kruer M. C., Kullmann D. M., Acevedo-Arozena A., Wells S. E., Corrochano S., Nolan P. M., Loss of *Frrs1l* disrupts synaptic AMPA receptor function, and results in neurodevelopmental, motor, cognitive and electrographical abnormalities. Dis. Model. Mech. 12, dmm036806 (2019). 30692144 10.1242/dmm.036806 PMC6398485

[35] Bourbia N., Chandler P., Codner G., Banks G., Nolan P. M., The guanine nucleotide exchange factor, *Spata13*, influences social behaviour and nocturnal activity. Mamm. Genome 30, 54–62 (2019). 31020388 10.1007/s00335-019-09800-9 PMC6491400

[36] Tucci V., Kleefstra T., Hardy A., Heise I., Maggi S., Willemsen M. H., Hilton H., Esapa C., Simon M., Buenavista M.-T., McGuffin L. J., Vizor L., Dodero L., Tsaftaris S., Romero R., Nillesen W. N., Vissers L. E. L. M., Kempers M. J., Vulto-van Silfhout A. T., Iqbal Z., Orlando M., Maccione A., Lassi G., Farisello P., Contestabile A., Tinarelli F., Nieus T., Raimondi A., Greco B., Cantatore D., Gasparini L., Berdondini L., Bifone A., Gozzi A., Wells S., Nolan P. M., Dominant β-catenin mutations cause intellectual disability with recognizable syndromic features. J. Clin. Invest. 124, 1468–1482 (2014). 24614104 10.1172/JCI70372 PMC3973091

[37] Douglas R. M., Alam N. M., Silver B. D., McGill T. J., Tschetter W. W., Prusky G. T., Independent visual threshold measurements in the two eyes of freely moving rats and mice using a virtual-reality optokinetic system. Vis. Neurosci. 22, 677–684 (2005). 16332278 10.1017/S0952523805225166

[38] Hardisty-Hughes R. E., Parker A., Brown S. D. M., A hearing and vestibular phenotyping pipeline to identify mouse mutants with hearing impairment. Nat. Protoc. 5, 177–190 (2010). 20057387 10.1038/nprot.2009.204

[39] Bains R. S., Cater H. L., Sillito R. R., Chartsias A., Sneddon D., Concas D., Keskivali-Bond P., Lukins T. C., Wells S., Arozena A. A., Nolan P. M., Armstrong J. D., Analysis of individual mouse activity in group housed animals of different inbred strains using a novel automated home cage analysis system. Front Behav. Neurosci. 10, 106 (2016). 27375446 10.3389/fnbeh.2016.00106 PMC4901040

[40] Risher W. C., Ustunkaya T., Singh Alvarado J., Eroglu C., Rapid Golgi analysis method for efficient and unbiased classification of dendritic spines. PLOS ONE 9, e107591 (2014). 25208214 10.1371/journal.pone.0107591 PMC4160288

[41] Ermolyuk Y. S., Alder F. G., Henneberger C., Rusakov D. A., Kullmann D. M., Volynski K. E., Independent regulation of basal neurotransmitter release efficacy by variable Ca^2+^ influx and bouton size at small central synapses. PLOS Biol. 10, e1001396 (2012). 23049481 10.1371/journal.pbio.1001396 PMC3457933

[42] Schmitz S. K., Hjorth J. J. J., Joemai R. M. S., Wijntjes R., Eijgenraam S., de Bruijn P., Georgiou C., de Jong A. P. H., van Ooyen A., Verhage M., Cornelisse L. N., Toonen R. F., Veldkamp W. J. H., Automated analysis of neuronal morphology, synapse number and synaptic recruitment. J. Neurosci. Methods 195, 185–193 (2011). 21167201 10.1016/j.jneumeth.2010.12.011

[43] Volynski K. E., Rusakov D. A., Kullmann D. M., Presynaptic fluctuations and release-independent depression. Nat. Neurosci. 9, 1091–1093 (2006). 16878129 10.1038/nn1746 PMC3433797

