# Supplementary material for: Forward genetics identifies a novel sleep mutant with sleep state inertia and REM sleep deficits
Source: Sci Adv. 2020 Aug 12;6(33):eabb3567. doi: 10.1126/sciadv.abb3567 (PMC7423362; doi:10.1126/sciadv.abb3567)
Supplement: abb3567_SM.pdf [file abb3567_SM.pdf]

Science Advances
AAAS

advances.sciencemag.org/cgi/content/full/6/33/eabb3567/DC1

Supplementary Materials for

Forward genetics identifies a novel sleep mutant with sleep state inertia and REM sleep deficits

Gareth T. Banks, Mathilde C. C. Guillaumin, Ines Heise, Petrina Lau, Minghui Yin, Nora Bourbia, Carlos Aguilar, Michael R. Bowl, Chris Esapa, Laurence A. Brown, Sibah Hasan, Erica Tagliatti, Elizabeth Nicholson, Rasneer Sonia Bains, Sara Wells, Vladyslav V. Vyazovskiy, Kirill Volynski, Stuart N. Peirson, Patrick M. Nolan*

*Corresponding author. Email: p.nolan@har.mrc.ac.uk

Published 12 August 2020, *Sci. Adv.* **6**, eabb3567 (2020)
DOI: 10.1126/sciadv.abb3567

The PDF file includes:

Figs. S1 to S8
Legend for table S1
Legend for movie S1

Other Supplementary Material for this manuscript includes the following:

(available at advances.sciencemag.org/cgi/content/full/6/33/eabb3567/DC1)

Table S1
Movie S1

Supplementary Materials

Table S1. Data analysis and statistics.

Movie S1. Home-cage behaviours in *rlss* mice. Four short video clips showing unusual home-cage behaviours in group-housed mice. Animals are tagged with RFID chips and housed three per cage. Three *rlss* males are co-housed in the first three clips while one rlss female (cyan tag) is housed with two wild-type littermates in the final clip. In the first clip, one of the males (blue tag) sleeps in an unusual position in the centre of the cage without forming a nest while the other two males are grooming excessively, bouts of grooming can extend for periods longer than 10 minutes.

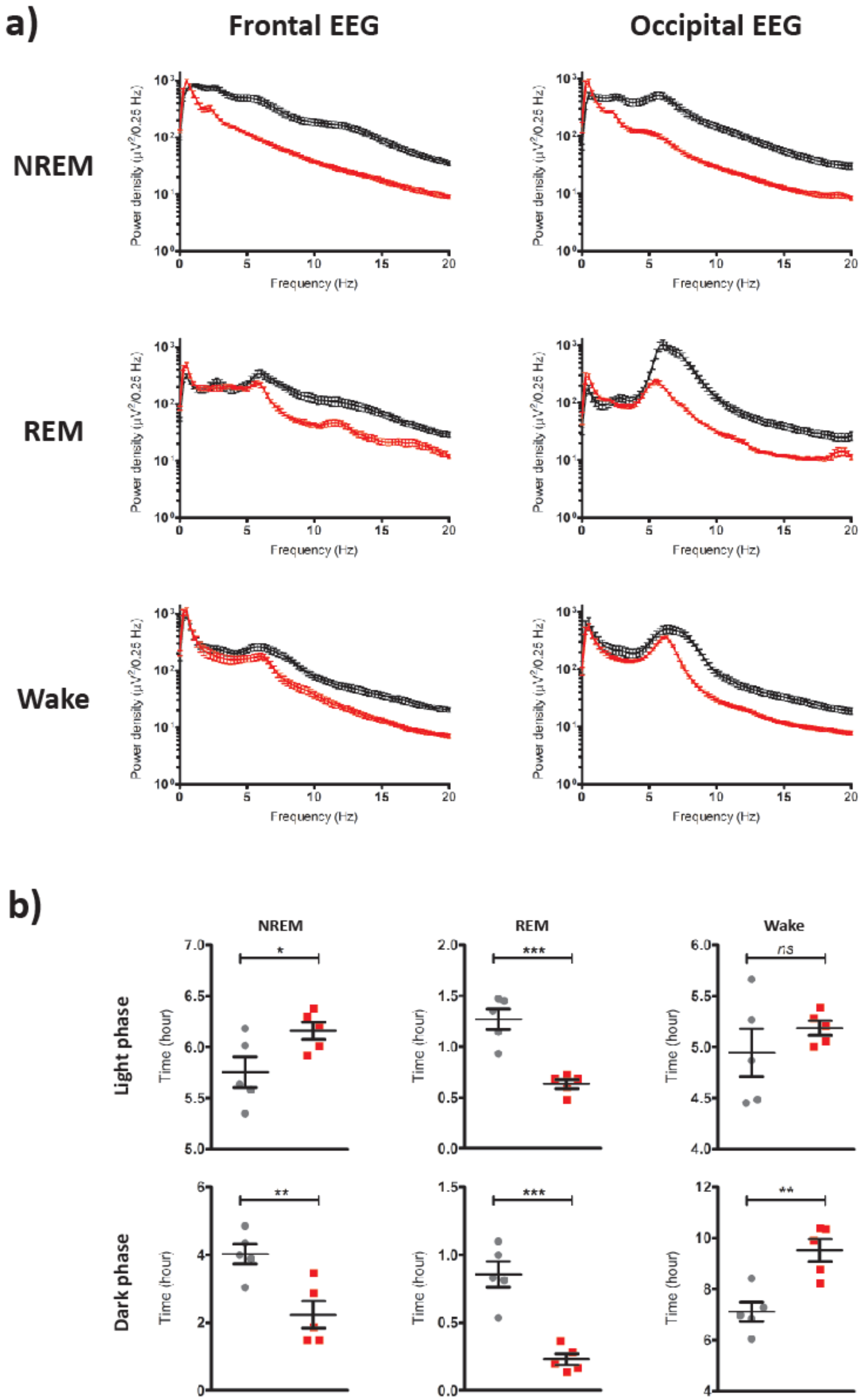

Fig. S1. a) Mean EEG power density spectra of the different vigilance states - wake, NREM and REM - in wild-type (black) and *Vamp2*^{rlss} (red) mice calculated from a 24-h baseline recording in the frontal (left panels) and occipital (right panels) derivations. **b)** Amount of time spent in each vigilance state during the light and dark phases of a baseline day in wild-type (grey) and *Vamp2*^{rlss} (red). mean ± SEM, *$p < 0.05$, **$p < 0.01$, ***$p < 0.001$.

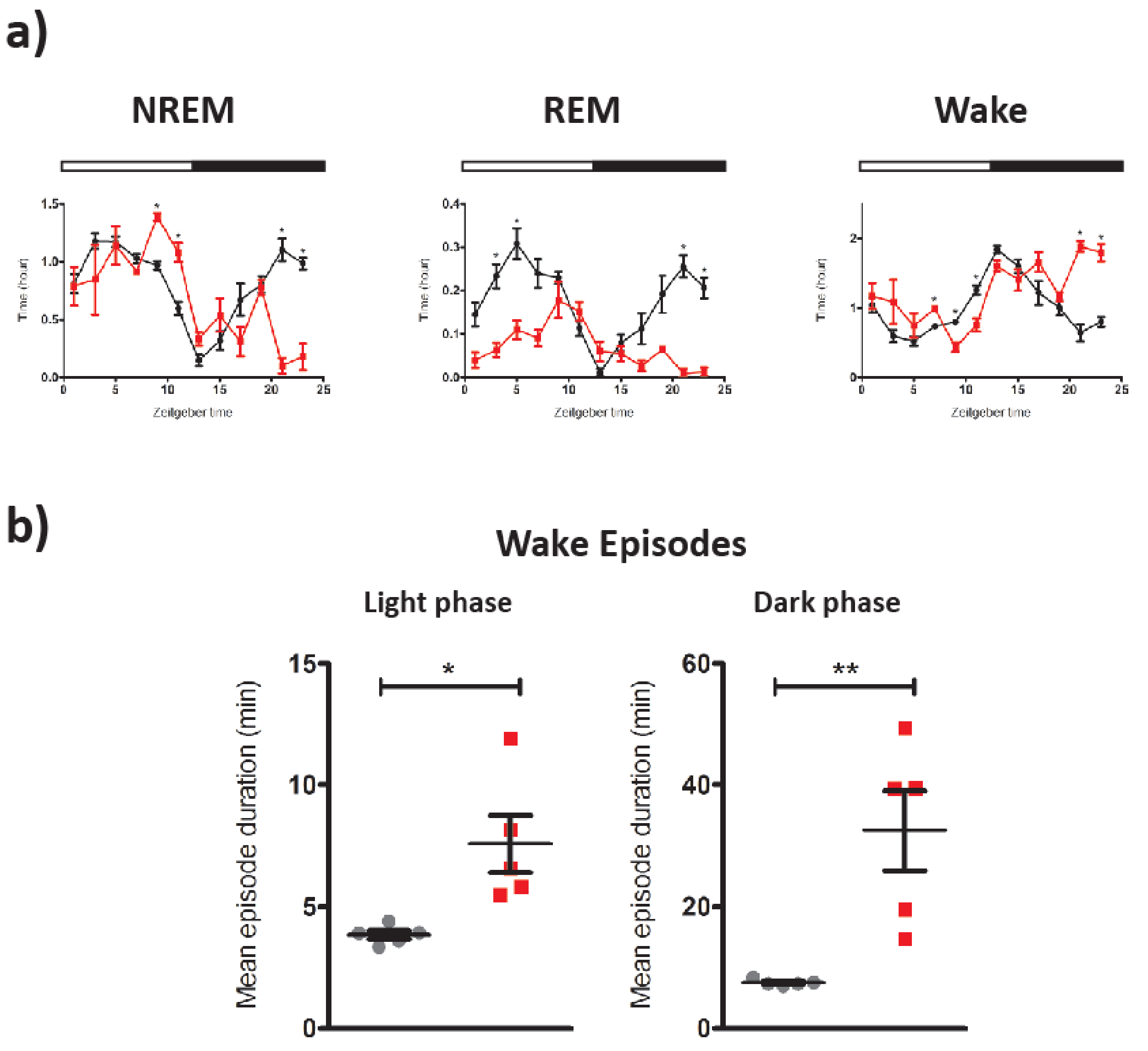

Fig. S2. a) Distribution of vigilance states across a 24-h baseline day in wild-type (black) and *Vamp2*^{rlss} (red) mice. Light and dark phases are indicated above each graph with white and black bars, respectively. Data are plotted in 2-h bins. **b)** Mean duration of wake episodes of at least 16 seconds (as opposed to 1 min in **Fig 2f**) during the 12-h light and dark phases of a baseline day. Mean ± SEM. *p < 0.05, **p < 0.01

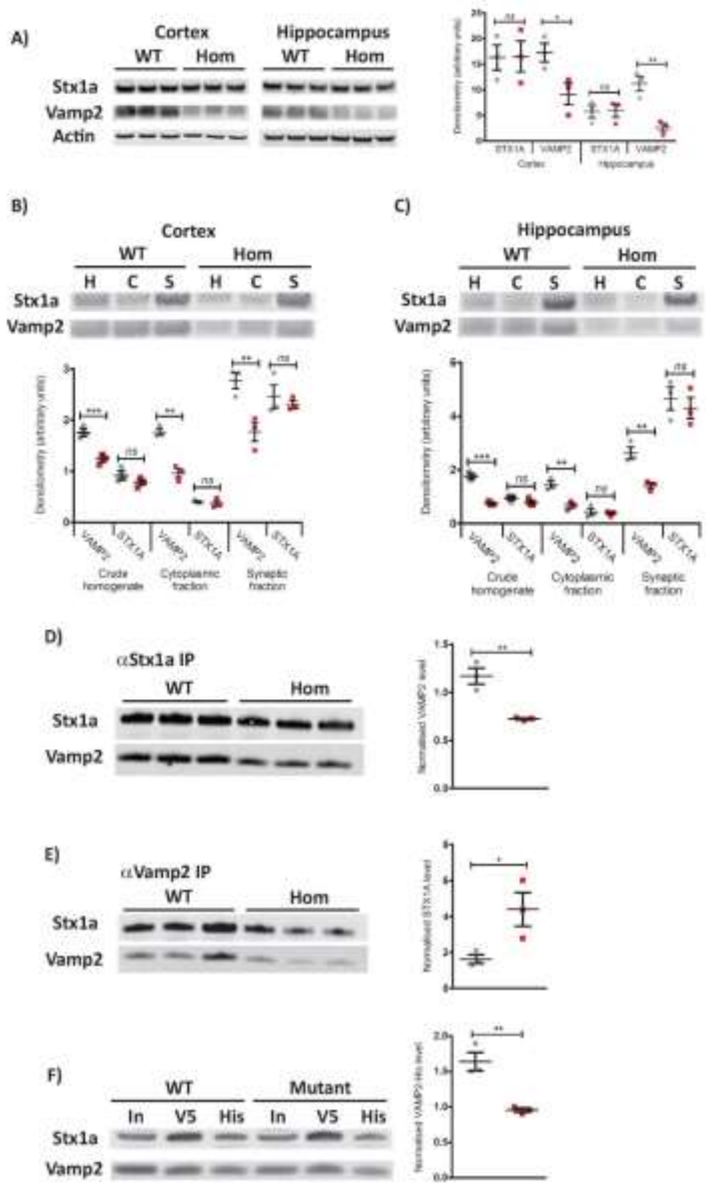

Fig. S3.

A) Western blots (left) and densitometry (right) of cortical and hippocampal whole cell lysates in wildtype (WT, grey) and *rlss* homozygous (Hom, red) samples. Levels of STX1a and VAMP2 are quantitated relative to actin levels. Western blots and densitometry of cortical **B)** and hippocampal **C)** whole cell (W), cytosolic (C) and synaptic (S) cellular fractions. Levels of STX1a and VAMP2 are quantitated relative to actin levels. Western blot and densitometry of IP from hippocampal tissue using either primary Stx1a **D)** or Vamp2 **E)** antibody. Levels of the co-precipitated protein are quantitated relative to the level of the primary antigen precipitated. **F)** Western blots and densitometry of pull downs using approximately equal levels of transfected 6xHis-VAMP2 (WT or mutant) and V5-STX1A proteins in HEK cells. Western blot shows result of a single set of pull downs showing input (In), precipitate using anti-V5 as the primary antibody (V5) and precipitate using anti-His as the primary antibody (His). Densitometry shows quantitation of the co-precipitate relative to the level of the primary antigen precipitated. Individual data points are shown (n=3) as is mean ± SEM, *$p < 0.05$, **$p < 0.01$, ***$p < 0.001$.

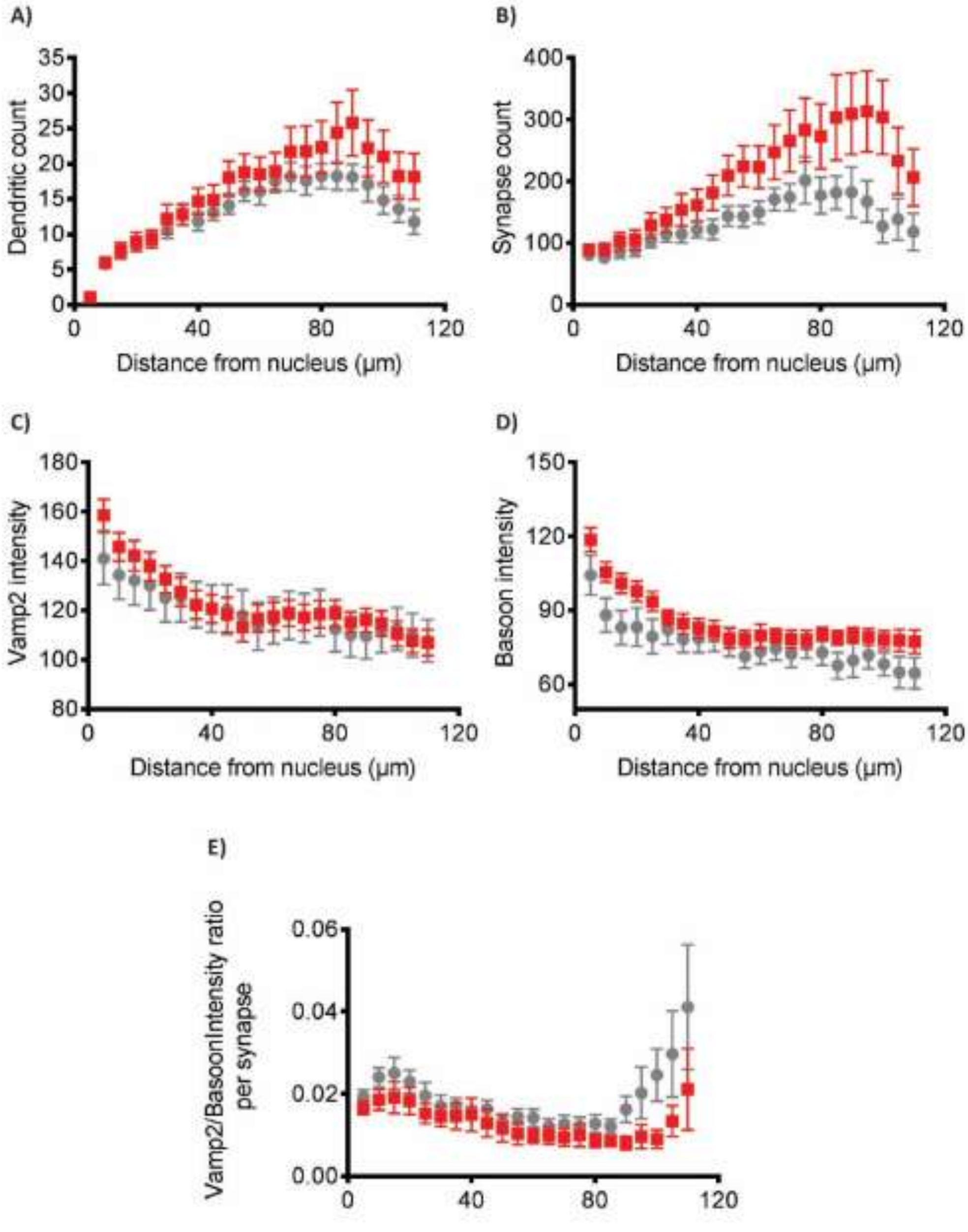

Fig. S4.

A) Dendritic branching in wildtype (grey) and mutant (red) cultures was evaluated by Sholl analysis of Map2 stained hippocampal neurons and dendritic branch counts were plotted against distance from the cell soma base. **B)** The number of pre-synaptic projections were evaluated using Bassoon as a marker and synaptic count plotted against distance from the cell soma base. **C)** Vamp2 immunofluorescence staining intensity plotted against distance from cell soma base. **D)** Bassoon immunofluorescence staining intensity plotted against distance from cell soma base. **E)** Vamp2 fluorescence intensity from **C)** normalized by Bassoon expression in **D)** in each synaptic projection from **B)**. Normalized Vamp2 expression was plotted against distance from soma base. Neurites were detected using SynD software. WT n=14, Hom n = 12. Data are expressed as mean ± SEM.

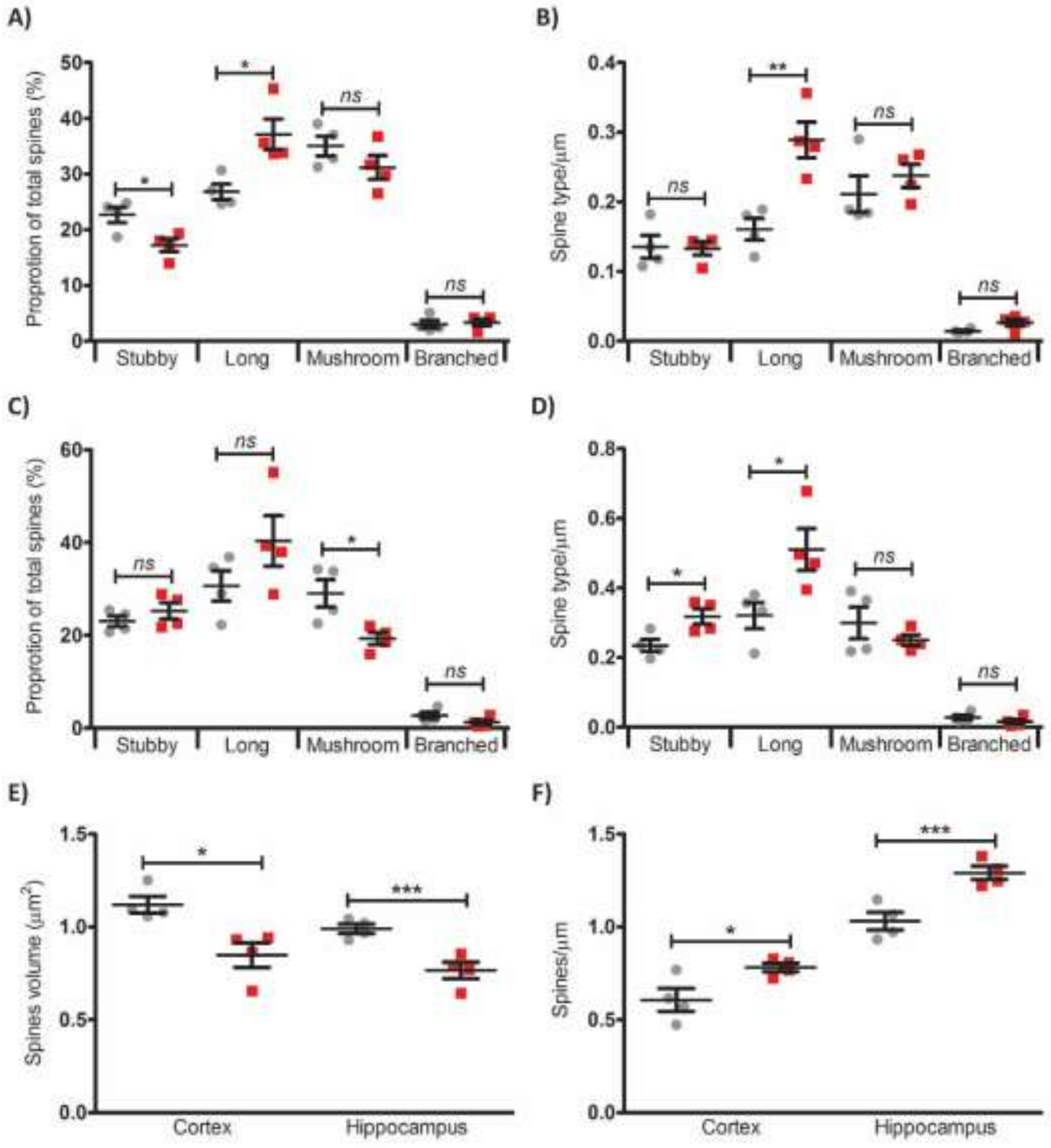

Fig. S5.

Golgi-Cox analysis of spines. Proportion and number of each spine type respectively in cortical (**A,B**) and hippocampal (**C,D**) sections from wildtype (grey) and *rlss* homozygous (red) mice. Average spine volume (**E**) and spine count per µm (**F**) in cortical and hippocampal sections. Individual data points are shown (n=4) as is mean ± SEM, *p < 0.05, **p < 0.01, ***p < 0.001.

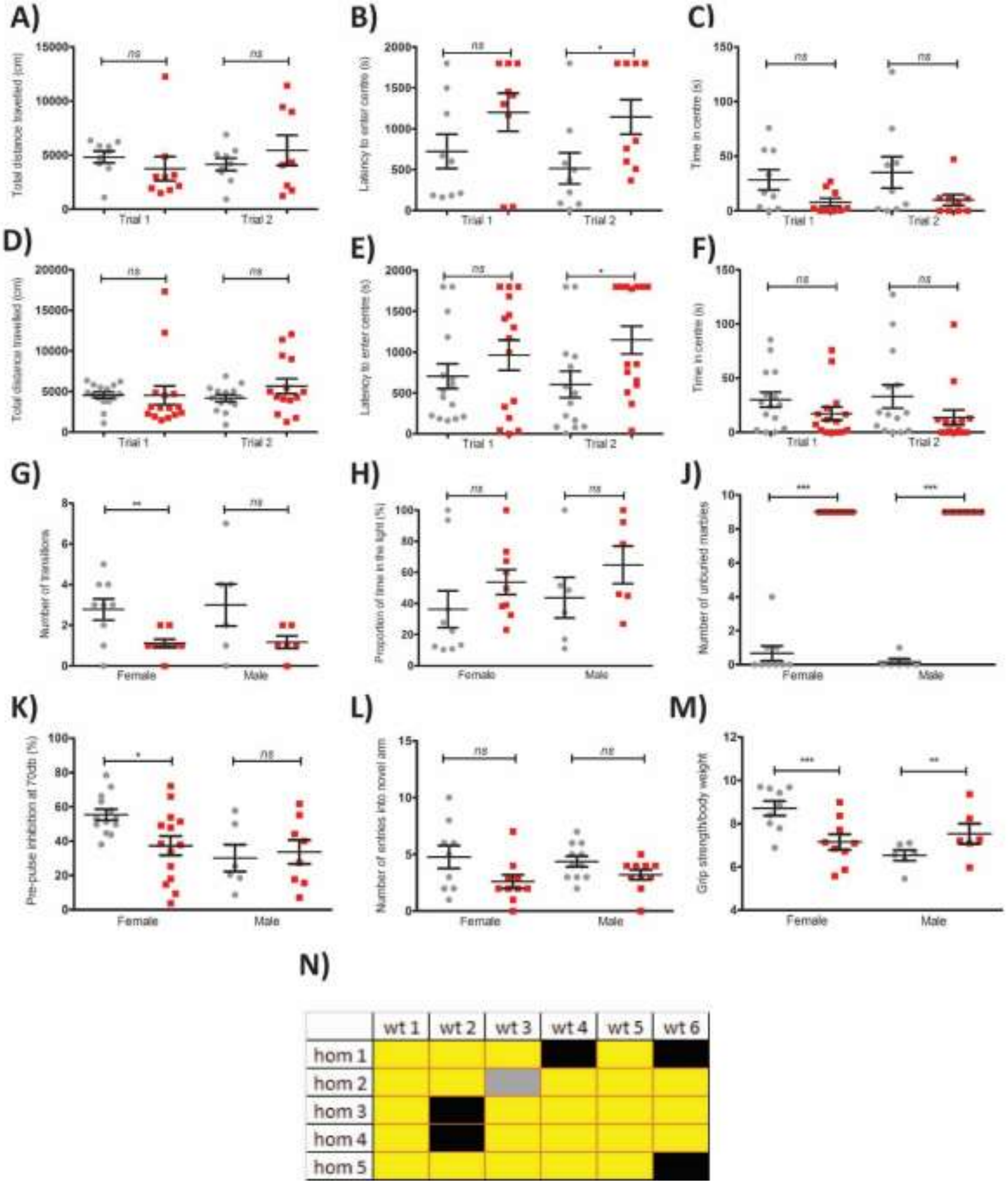

Fig. S6.

Behavioural test battery measures in wildtype (grey) and *rlss* homozygous (red) mice. Open field distance travelled, latency to centre area and time spent in centre for females (**A,B,C** respectively) and males (**D,E,F** respectively), **G**) number of transitions in a Light-dark box, **H**) proportion of time in dark compartment of a Light-dark box, **J**) numbers of marbles left unburied in marble burying test, **K**) Prepulse inhibition of acoustic startle response, **L**) Y-maze number of entries into novel arm, **M**) Grip strength adjusted to body weight, **N**) Social dominance tube test. Yellow = WT is dominant. Black =inconclusive. Grey = untested. Cohort numbers for individual tests are as shown. Mean ± SEM, $^{**}p < 0.01$, $^{***}p < 0.001$.

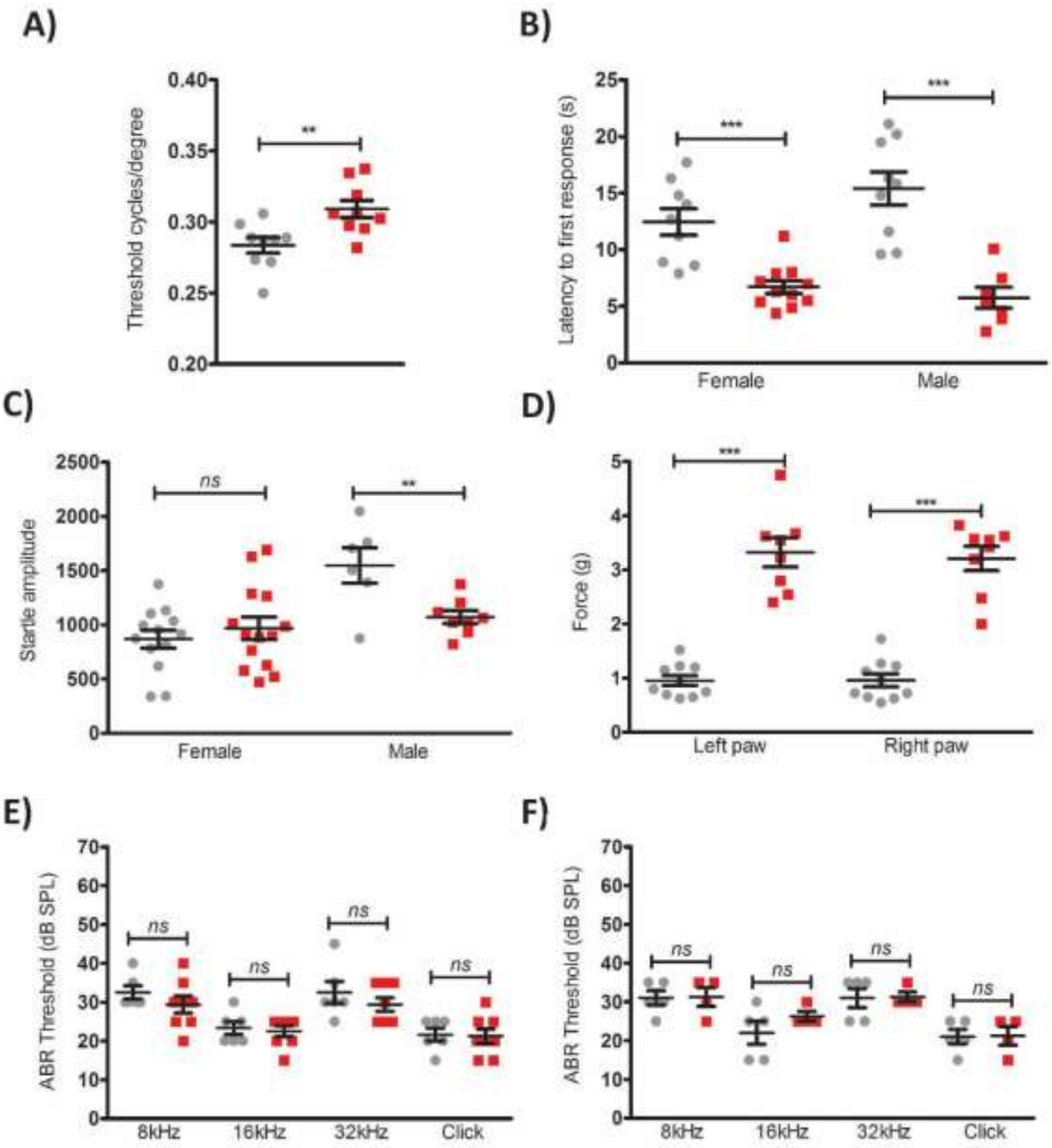

Fig. S7.

Sensory function in wildtype (grey) and *rlss* homozygous (red) mice. **A**) Visual acuity measured using an optokinetic response drum, **B**) Thermal nociception measuring latency to first response in hot plate test, **C**) Acoustic startle response, **D**) Mechanical sensitivity thresholds using the von Frey test, Acoustic brainstem response (ABR) threshold measures in males **E**) and females **F**). Cohort numbers for individual tests are as shown. Mean $\pm$ SEM, $^{**}p < 0.01$, $^{***}p < 0.001$.

A)

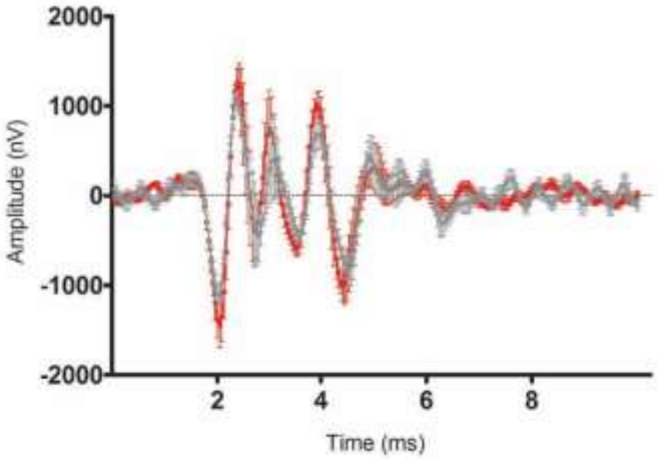

B)

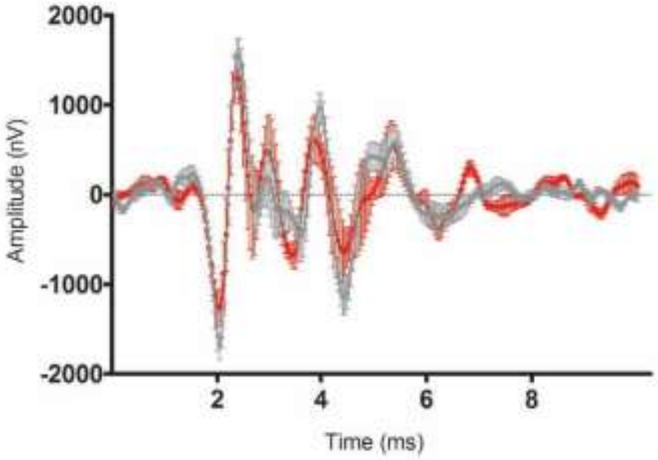

Fig. S8.

ABR waveform in response to a 70db stimulus in male **(A)** and female **(B)** animals showed no evidence of synaptic malfunction along the auditory pathway. There are no overall differences in the amplitude or latency of ABR components of mutant mice compared to wildtype controls suggesting no likely malfunction of the various peak-generating synapses along the auditory pathway. Traces show mean waveform ± SEM (Male WT, n=5; Male Hom, n=4; Female WT, n=6; Female Hom, n=8).
